# On Quantization Errors in Approximate and Sample Entropy

**DOI:** 10.3390/e24010073

**Published:** 2021-12-31

**Authors:** Dragana Bajić, Nina Japundžić-Žigon

**Affiliations:** 1 Faculty of Technical Sciences, Department of Communications and Signal Processing, University of Novi Sad, Trg Dositeja Obradovica 6, 21000 Novi Sad, Serbia; 2 Faculty of Medicine, University of Belgrade, dr Subotića 8, 11000 Belgrade, Serbia; nzigon@med.bg.ac.rs

**Keywords:** approximate entropy, sample entropy, quantization error, matching probabilities, interpolation

## Abstract

Approximate and sample entropies are acclaimed tools for quantifying the regularity and unpredictability of time series. This paper analyses the causes of their inconsistencies. It is shown that the major problem is a coarse quantization of matching probabilities, causing a large error between their estimated and true values. Error distribution is symmetric, so in sample entropy, where matching probabilities are directly summed, errors cancel each other. In approximate entropy, errors are accumulating, as sums involve logarithms of matching probabilities. Increasing the time series length increases the number of quantization levels, and errors in entropy disappear both in approximate and in sample entropies. The distribution of time series also affects the errors. If it is asymmetric, the matching probabilities are asymmetric as well, so the matching probability errors cease to be mutually canceled and cause a persistent entropy error. Despite the accepted opinion, the influence of self-matching is marginal as it just shifts the error distribution along the error axis by the matching probability quant. Artificial lengthening the time series by interpolation, on the other hand, induces large error as interpolated samples are statistically dependent and destroy the level of unpredictability that is inherent to the original signal.

## 1. Introduction

Despite the global spread of mobile phones and multimedia, few people know that digital technology is based on the work of one scientist—Claude Elwood Shannon. His work on the sampling theorem [1], channel capacity and compression [1,2], cryptography [3], and coding [4] initiated a new scientific discipline—information theory [5].

One of the key findings was a measure of the information uncertainty of the communication signal [2]. It was called “entropy”, at the suggestion of J. von Neumann [6], following a similar measure from the field of classical thermodynamics. A generalization suitable for dynamical systems was introduced in [7] but theoretically too demanding for practical application. With the relaxation of mathematical constraints first by the approximate entropy (*ApEn*, [8]) and then by its modification sample entropy (*SampEn*, [9]), entropy began to break into other areas, with the growing popularity, and with citations measuring in the thousands [10].

Biomedical sciences are among the leaders in finding new applications as an area that offers one of the largest selections of diverse signals to be analyzed. A nonlinear regularity metric enabled by *ApEn* and *SampEn* might reveal the physiological mechanisms that would otherwise remain hidden. Just to mention a very few applications, entropy was implemented for medical image segmentation [11], melanoma detection [12], in neurosciences [13], for heart rate in healthy subjects [14], during sporting activities [15], in patients with heart failure [16], or with diabetes [17].

*ApEn* and *SampEn* are subject to several levels of freedom: the time series length *N* and the threshold *r*—the similarity criterion that defines (un)predictability within the time series, the length of the pattern vector (similarity) m, and the time delay *τ*. Their incorrect application can lead to different results within the same experimental settings (so-called “flip-flop” effect) [18,19]. Detailed parameter studies are numerous [13,20,21,22,23,24,25,26,27,28,29], with one of the most thorough analyses given recently in [30]. However, studies devoted to particular biomedical problems prefer to perform a quick search and find the parameters tuned to the signal they investigate. Thus far, guidelines or recommendations on implementation methods do not exist, despite the extensive application.

Sampling frequency has recently come into focus as another potential level of freedom. In [31], *SampEn* was estimated from source signals (hip, knee, and jump angle in the sagittal plane), reporting that an increase in sampling frequency significantly reduced *SampEn*. Similar results are shown in [32] (box-plots), representing the *SampEn* of the original ECG and EEG signals. The tables in [33] show the same *SampEn* reduction with sampling frequency, where *SampEn* was applied to the main atrial wave (MAV) waveforms derived from the original ECG signal and then upsampled or downsampled to mimic different sampling frequencies.

These results are expected. The lowest sampling frequency has to follow theoretical requirements. Sampling frequency should be twice the maximal spectral component of the observed signal [2], providing sufficient samples for its unambiguous reconstruction. Increased sampling frequency provides redundant, and therefore statistically dependent, samples, thus attenuating the unpredictability of the observed signal. For this reason, the entropy of oversampled signals decreases.

The purpose of this paper is to examine the causes of entropy estimation errors underlying deficiencies such as bias, relative inconsistency, and sample length dependence. In addition, it is sometimes difficult to meet the minimum signal length requirements [20], so artificially extending the signal by interpolating [34] might cause an additional error. Knowing why errors occur is a safe way to find a method to combat them. For this reason, we analyzed the entropy of artificial signals with symmetric and asymmetric distributions, long time series of pulse intervals recorded from laboratory rats, and shorter heart rate signals recorded from healthy volunteers in an outpatient setting.

## 2. Materials and Methods

### 2.1. Approximate and Sample Entropy in Brief

The entropy analysis begins with the segmentation of the time series *x_i_* ∈ **X**, *i* = 1, …, *N*, into the overlapping vectors of length *m*:

(1) $$X_{i}^{\left(m\right)}=\left[x_{i},x_{i+\tau },\cdots ,x_{i+\left(m-1\right)\cdot \tau }\right],\;\;i=1,\cdots ,N-\left(m-1\right)\cdot \tau .$$

The parameter *τ* separates the signal samples, thus decorrelating them [29]. Most applications assume that the vectors are composed of adjacent samples and *τ* > 1 is not common, so we adopted *τ* = 1.

The *ApEn* and *SampEn* procedures are based on counting similar vectors. A criterion for similarity is a condition that maximal absolute distance between the scalar components of two vectors $X_{i}^{\left(m\right)}$ and $X_{j}^{\left(m\right)}$ should be below some predefined threshold *r*:

(2) $$d\left(X_{i}^{\left(m\right)},X_{j}^{\left(m\right)}\right)=\underset{k=0,\cdots ,m-1}{\max}\left|x_{i+k}-x_{j+k}\right|\leq r,\;\;\;i,j=1,\cdots ,N-m+1$$

The observed vector $X_{i}^{\left(m\right)}$ is “*template*”, and the vectors $X_{j}^{\left(m\right)},\;j=1,\ldots ,N-m+1$ that satisfy condition (2) are its “*matches*”. Comparison of the template to itself is “*self-matching*” and occurs in *ApEn*. The absence of any matches is “*zero-match*” and occurs only in *SampEn*. The number of matches in *ApEn* and *SampEn* is not equal, as *SampEn* excludes both self-matches (*j* = *i*) and the last comparison (*j* = *N* − *m* + 1):

$$C_{i}^{\left(m\right)}\left(r\right)=\sum_{j=1}^{N-m+1}I\left\{d\left(X_{i}^{\left(m\right)},X_{j}^{\left(m\right)}\right)\leq r\right\},\;\;i=1,\cdots ,N-m+1,\;\;\mathrm{for}\;ApEn;$$

(3) $$B_{i}^{\left(m\right)}\left(r\right)=\sum_{j=1,j\neq i}^{N-m}I\left\{d\left(X_{i}^{\left(m\right)},X_{j}^{\left(m\right)}\right)\leq r\right\},\;\;i=1,\cdots ,N-m,\;\;\mathrm{for}\;SampEn;$$

The sum of indicator functions from (3), ∑I{condition}, describes the counting process, as the indicator function is equal to one of the *condition* it indicates is fulfilled; otherwise, it is equal to zero. The notations of the number of matches are from [8,9].

The number of matches divided by the number of tests gives a classical definition of probability as a relative frequency. The probability that the template vector will find its match (“*matching probability*”) is estimated as:

$${\hat{p}}_{Ai}^{\left(m\right)}\left(r\right)=\frac{C_{i}^{\left(m\right)}\left(r\right)}{N-m+1},\;\;i=1,\cdots ,N-m+1,\;\;\mathrm{for}\;ApEn;$$

(4) $${\hat{p}}_{Si}^{\left(m\right)}\left(r\right)=\frac{B_{i}^{\left(m\right)}\left(r\right)}{N-m},\;\;i=1,\cdots ,N-m,\;\;\mathrm{for}\;SampEn,$$

where $“\hat{p}”$ denotes an estimate od *p*.

*ApEn* and *SampEn* also require matching probability estimates for *m* + 1 templates:

$${\hat{p}}_{Ai}^{\left(m+1\right)}\left(r\right)=\sum_{j=1}^{N-m}I\left\{d\left(X_{i}^{\left(m+1\right)},X_{j}^{\left(m+1\right)}\right)\leq r\right\}=\frac{C_{i}^{\left(m+1\right)}\left(r\right)}{N-m},\;\;i=1,\cdots ,N-m,\;\;\mathrm{for}\;ApEn;$$

(5) $${\hat{p}}_{Si}^{\left(m+1\right)}\left(r\right)=\sum_{j=1,j\neq i}^{N-m}I\left\{d\left(X_{i}^{\left(m+1\right)},X_{j}^{\left(m+1\right)}\right)\leq r\right\}=\frac{C_{i}^{\left(m+1\right)}\left(r\right)-1}{N-m}=\frac{A_{i}^{\left(m\right)}\left(r\right)}{N-m},\;\;i=1,\cdots ,N-m,\;\mathrm{for}\;SampEn;$$

where $A_{i}^{\left(m\right)}\left(r\right)$ is the number of matches for vectors of length *m* + 1 (notation from [9]).

The source papers on *ApEn* and *SampEn* did not include matching probabilities, but only the number of matching vectors $A_{i}^{\left(m\right)}\left(r\right)$, $B_{i}^{\left(m\right)}\left(r\right)$, and $C_{i}^{\left(m\right)}\left(r\right)$ defined in (3) and (5). We opted to work with probabilities as it is mathematically more palatable to operate with the probabilities that two vectors match than with the number of matching vectors.

Then the *ApEn* estimate is defined as:

(6) $$\hat{A}pEn\left(m,r,N\right)={\;\hat{\Phi }}^{\left(m\right)}\left(r,N\right)-{\;\hat{\Phi }}^{\left(m+1\right)}\left(r,N\right)$$

where

(7) $${\hat{\Phi }}^{\left(m\right)}\left(r,N\right)=\frac{1}{N-m+1}\cdot \sum_{i=1}^{N-m+1}\ln\left({\hat{p}}_{Ai}^{\left(m\right)}\left(r\right)\right),$$

and “ln” is the natural logarithm.

Similarly, *SampEn* estimate is defined as:

(8) $$\hat{S}ampEn\left(m,r,N\right)=-\ln\left(\frac{\sum_{j=1,j\neq i}^{N-m}A_{i}^{\left(m\right)}\left(r\right)}{\sum_{j=1,j\neq i}^{N-m}B_{i}^{\left(m\right)}\left(r\right)}\right)={\;\hat{\Psi }}^{\left(m\right)}\left(r,N\right)-{\;\hat{\Psi }}^{\left(m+1\right)}\left(r,N\right)$$

where

$${\;\hat{\Psi }}^{\left(m\right)}\left(r,N\right)=\ln\left(\frac{1}{N-m}\cdot \sum_{j=1,j\neq i}^{N-m}B_{i}^{\left(m\right)}\left(r\right)\right)=\ln\left(\frac{1}{N-m}\cdot \sum_{j=1,j\neq i}^{N-m}{\hat{p}}_{Si}^{\left(m\right)}\left(r\right)\right),$$

(9) $${\hat{\Psi }}^{\left(m+1\right)}\left(r,N\right)=\ln\left(\frac{1}{N-m}\cdot \sum_{j=1,j\neq i}^{N-m}{\hat{p}}_{Si}^{\left(m+1\right)}\left(r\right)\right).$$

### 2.2. Errors in Entropy Estimation

The core of *ApEn* and *SampEn* estimates is the probability that the template vector is similar to other vectors. As with any probability, it is prone to estimation errors. Such errors are analyzed in detail in [35], in the data communication environment, for binary transmission errors. However, entropy encompasses the sum of many probabilities, and their impact on the overall result is not straightforward.

The estimated probability ${\hat{p}}_{Zi}^{\left(m\right)}\left(r\right)$, where Z∈{A,S} is included to denote both *ApEn* and *SampEn*, can be expressed as a difference of the correct (calculated) matching probability value $p_{i}^{\left(m\right)}\left(r\right)$, which is the same for both entropies and error $\varepsilon_{Zi}^{\left(m\right)}\left(r\right)$:

(10) $${\hat{p}}_{Zi}^{\left(m\right)}\left(r\right)=p_{i}^{\left(m\right)}\left(r\right)-\varepsilon_{Zi}^{\left(m\right)}\left(r\right),\;\;Z\in \left\{A,S\right\}$$

The impact of errors on the summand ${\hat{\Phi }}^{\left(m\right)}\left(r,N\right)$ is as follows:

(11) $${\hat{\Phi }}^{\left(m\right)}\left(r,N\right)=\frac{1}{N-m+1}\cdot \sum_{i=1}^{N-m+1}\ln\left(p_{i}^{\left(m\right)}\left(r\right)-\varepsilon_{Ai}^{\left(m\right)}\left(r\right)\right)=\frac{1}{N-m+1}\cdot \sum_{i=1}^{N-m+1}\ln\left(p_{i}^{\left(m\right)}\left(r\right)\cdot \left(1-\frac{\varepsilon_{Ai}^{\left(m\right)}\left(r\right)}{p_{i}^{\left(m\right)}\left(r\right)}\right)\right)=\;\;=\frac{1}{N-m+1}\cdot \sum_{i=1}^{N-m+1}\ln\left(p_{i}^{\left(m\right)}\left(r\right)\right)+\frac{1}{N-m+1}\cdot \sum_{i=1}^{N-m+1}\ln\left(1-\frac{\varepsilon_{Ai}^{\left(m\right)}\left(r\right)}{p_{i}^{\left(m\right)}\left(r\right)}\right)=\Phi^{\left(m\right)}\left(r,N\right)+\varepsilon_{\Phi }^{\left(m\right)}\left(r,N\right).$$

Similarly, for ${\hat{\Psi }}^{\left(m\right)}\left(r,N\right)$:

(12) $${\hat{\Psi }}^{\left(m\right)}\left(r,N\right)=\ln\left(\sum_{i=1}^{N-m}\left(p_{i}^{\left(m\right)}\left(r\right)-\varepsilon_{Ai}^{\left(m\right)}\left(r\right)\right)\right)=\ln\left(\left(\sum_{i=1}^{N-m}p_{i}^{\left(m\right)}\left(r\right)\right)\cdot \left(1-\frac{\sum_{i=1}^{N-m}\varepsilon_{Ai}^{\left(m\right)}\left(r\right)}{\sum_{i=1}^{N-m}p_{i}^{\left(m\right)}\left(r\right)}\right)\right)\;\;=\ln\left(\sum_{i=1}^{N-m}p_{i}^{\left(m\right)}\left(r\right)\right)+\ln\left(1-\frac{\sum_{i=1}^{N-m}\varepsilon_{Ai}^{\left(m\right)}\left(r\right)}{\sum_{i=1}^{N-m}p_{i}^{\left(m\right)}\left(r\right)}\right)=\Psi^{\left(m\right)}\left(r,N\right)+\varepsilon_{\Psi }^{\left(m\right)}\left(r,N\right)$$

It follows that the estimation errors for *ApEn* and *SampEn* can be expressed as:

(13) $$\varepsilon_{ApEn}^{\left(m\right)}\left(r,N\right)=\varepsilon_{\Phi }^{\left(m\right)}\left(r,N\right)-\varepsilon_{\Phi }^{\left(m+1\right)}\left(r,N\right)\;\mathrm{and}\;\varepsilon_{SampEn}^{\left(m\right)}\left(r,N\right)=\varepsilon_{\Psi }^{\left(m\right)}\left(r,N\right)-\varepsilon_{\Psi }^{\left(m+1\right)}\left(r,N\right)$$

In order to implement the error analysis, besides the values estimated from the time series ((3)–(5)), it is also necessary to know the exact values of matching probabilities $p_{i}^{\left(m\right)}\left(r\right)$ for each template.

If the probability density function of the observed time series is $f_{x}\left(x\right)$ and following the similarity criterion $\left|x_{i}-x_{j}\right|\leq r$, the exact probability that the sample *x_i_* ∈ **X** will find a similar sample is equal to:

(14) $$p_{i}\left(r\right)=\int_{x_{i}-r}^{x_{i}+r}f_{x}\left(x\right)\cdot dx,\;\;i=1,\cdots ,N$$

The template vector of length *m* will find its match if its *k*th sample is similar to the *k*th sample of the matching vector for all *m* template samples. The exact matching probability can be calculated for all the template vectors of the time series:

(15) $$p_{i}^{\left(m\right)}\left(r\right)=\prod_{k=0}^{m-1}\int_{x_{i}-r}^{x_{i}+r}f_{x}\left(x\right)\cdot dx,\;\;\;\;i=1,\cdots ,N-m\;\left(\mathrm{or}\;N-m+1\right)$$

With the estimated and calculated matching probabilities, it is possible to estimate all the elements for error analysis, (10)–(13), as well as to calculate the entropy of the time series. It should be noted that the definition $\varepsilon_{\Phi }^{\left(m\right)}\left(r,N\right)=\frac{1}{N-m+1}\cdot \sum_{i=1}^{N-m+1}\ln\left(1-\frac{\varepsilon_{Ai}^{\left(m\right)}\left(r\right)}{p_{i}^{\left(m\right)}\left(r\right)}\right)$ (11) is formal, as the estimation error $\varepsilon_{Ai}^{\left(m\right)}\left(r\right)$ can exceed the true probability value $p_{i}^{\left(m\right)}\left(r\right)$ so a logarithm of negative value may occur. However, ${\hat{\Phi }}^{\left(m\right)}\left(r,N\right){\;\mathrm{and}\;\Phi }^{\left(m\right)}\left(r,N\right)$ are always defined, so the estimation error $\varepsilon_{\Phi }^{\left(m\right)}\left(r,N\right)={\hat{\Phi }}^{\left(m\right)}\left(r,N\right)-\Phi^{\left(m\right)}\left(r,N\right)$ always exists. Contrary to this, $\sum_{j=1,j\neq i}^{N-m}{\hat{p}}_{Si}^{\left(m\right)}\left(r\right)$ is a sum of the probabilities that exclude self-matches, and this sum can be equal to zero. In this case, summands ${\hat{\Psi }}^{\left(m\right)}\left(r,N\right)$, (9), as well as the corresponding *SampEn*, (8), are not defined.

The problem with this approach is that the exact probability distribution functions of the biomedical time series are not available. For this reason, we first performed an error analysis using artificially generated time series with known distributions. Then, based on these results, we analyzed the entropy estimated from the experimental signals.

### 2.3. Artificial and Experimental Data

In order to find the error in estimating entropy, we need a reference (correct) value of entropy. Artificially generated data with a known probability density function (PDF) provide this capability. If the PDF is known, the true entropy values can be calculated. On the other hand, all-purpose software provides the ability to generate a time series of arbitrary length *N* with signal samples that follow the same distribution. The entropy estimated from such a time series, compared with its true calculated value, shows the error in entropy estimation.

The artificially generated data we used comprise time series from three distributions chosen to show the impact of bounded, unbounded, and skewed data. An example of bounded and symmetric distribution is a uniform distribution. An example of symmetrical unbounded distribution is Gaussian, while skewed distribution is exponential. Additional property is that the corresponding integrals can be expressed either in a closed form or using the common built-in functions. In order to have errors of comparable amplitude, we performed standard scaling, i.e., we normalized and centralized the variables. Such scaling also avoids the necessity to adjust the threshold *r* to the signal standard deviation.

The following PDFs were used to generate the time series:

$$f_{x}^{\left(U\right)}\left(x\right)=\{\begin{array}{c} {\left(2\cdot \sqrt{3}\right)}^{-1},\;\;\;\left|x\right|\leq \sqrt{3}\\ 0,\;\;\;\mathrm{elsewhere} \end{array}$$

$$f_{x}^{\left(G\right)}\left(x\right)={\left(\sqrt{2\cdot \pi }\right)}^{-1}e^{-x^{2}/2},\;\forall x$$

(16) $$f_{x}^{\left(E\right)}\left(x\right)=e^{-x-1},\;\;x\geq -1$$

**Figure 1:**
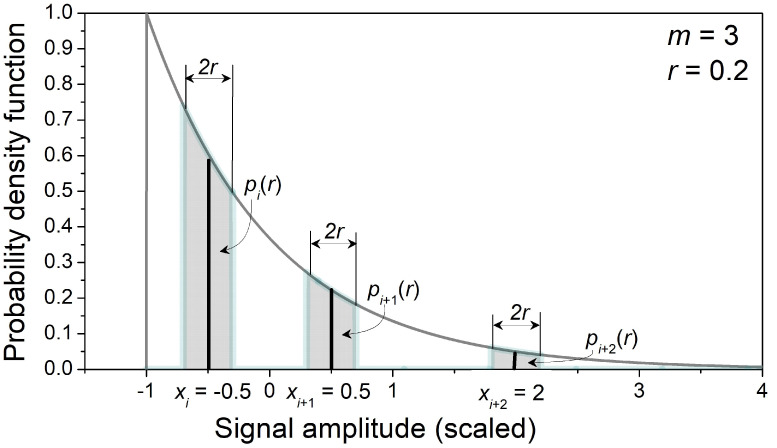
An example of a template vector $\left[\begin{array}{ccc} x_{i} & x_{i+1} & x_{i+2} \end{array}\right]=\left[\begin{array}{ccc} -0.5 & 0.5 & 2 \end{array}\right]$ and the corresponding calculated matching probabilities $p_{i}\left(r\right),\;p_{i+1}\left(r\right),\;$ and $p_{i+2}\left(r\right),\;$ equal to the shaded areas of PDF. In this example, *m* = 3, *r* = 0.2, and samples are from a normalized and centralized exponential distribution.

The time series were generated using built-in PDF generators, and the generated distributions were checked using the Kolmogorov–Smirnov test [36]. The probabilities ${\hat{p}}_{Zi}^{m}\left(r\right)$ were estimated by the counting process, according to (3)–(5). The probabilities $p_{i}\left(r\right)$ are calculated for each sample of the template (Figure 1) according to (14):

(17) $$p_{i}\left(r\right)=\{\begin{array}{c} \frac{r\;+\;\min\left(r,\sqrt{3}\;-\;\left|x_{i}\right|\right)}{\left(2\cdot \sqrt{3}\right)},\;\;\;\mathrm{uniform}\\ \frac{\mathrm{erf}\left(\frac{\left(x_{i}\;+\;r\right)}{\sqrt{2}}\right)\;-\;\mathrm{erf}\left(\frac{\left(x_{i}\;-\;r\right)}{\sqrt{2}}\right)}{2},\;\mathrm{Gaussian}\\ e^{-1-x_{i}+r}-e^{-1-x_{i}-r},\;\;\;\;\mathrm{exponential} \end{array},$$

with a built-in function $\mathrm{erf}\left(a\right)=\frac{2}{\sqrt{\pi }}\int_{0}^{a}e^{-x^{2}}\cdot dx$. The probabilities of *m* adjacent samples are then included in (15) to calculate $p_{i}^{\left(m\right)}\left(r\right)$.

After obtaining both estimated and calculated matching probabilities, all the other parameters can be found. Additionally, in the case of uniform distribution, the entropy can be calculated in a closed form. The expression is the same both for *ApEn* and *SampEn*:

(18) $$ApEn\left(r\right)=SampEn\left(r\right)=-ln\left(\frac{4\cdot r\cdot \sqrt{3}-r^{2}}{12}\right)$$

The derivation is simple, and it is given in the Appendix of [37].

The experimental time series were recorded from adult male conscious and unrestrained Wistar outbred rats, weighing 300–350 g, housed separately under control laboratory conditions (temperature −22 ± 2 °C; relative humidity: 60–70%; lighting: 12:12 h light–dark cycle) with food and water ad libitum.

The measurement of blood pressure waveforms (BP) was performed using TL11M2-PA-C50-PX-DSI equipment implanted into the abdominal aorta. The sampling frequency was 1000 Hz, with a 12-bit A/D converter yielding 4096 amplitude levels. We chose the signals from a pharmacological experiment [38] (baseline condition), for which data acquisition lasted more than an hour. The experiment provided a very long time series that allowed testing the effect of variable signal length on entropy estimation errors. Systolic blood pressure (SBP) and pulse interval (PI) time series were derived from the BP waveforms as the local maxima and as the intervals between the successive maximal BP positive changes, respectively. Artifacts were detected semi-automatically [39], with residual errors removed manually. The time series were detrended to remove the very slowly varying signal components by a filter proposed for biomedical applications in [40]. Each data segment was checked for stationarity both in mean and in variance by a method from [41].

All experimental procedures conformed to Directive 2010/63/EU national Animal Welfare Act 2009/6/RS and Rule Book 2010/RS on the protection of animals used for scientific purposes, insisting on the minimal number of experimental subjects. The protocol was approved by the University of Belgrade Ethics review board (license n°323-07-10519/2013-05/2).

Data series from human subjects were recorded from 77 medically examined male healthy volunteers in ideal hospital conditions, using Task Force monitor. Data lengths were typical for ambulatory monitoring, up to 10 min, and ensured the tranquility of the patient and stationarity of the recorded data. Artifacts were removed, and stationarity was checked in the same way as for the previous experimental time series. The experiment followed the ethical standards stated by the School of Medicine, University of Belgrade, in accordance with the Declaration of Helsinki, and the protocol was approved by the Ethics Committee of University Hospital Center Bežanijska Kosa, Belgrade, Serbia, No. 11754/3 from December 2015 for project TR32040. Signed permission was collected from each volunteer.

### 2.4. The Impact of Interpolation Errors

Medical recordings in patients might be very short. Some researchers limit the observed signals to 1 min, providing on average 75 beat-to-beat samples in human subjects. However, the source papers on entropy [8,9,20] pointed to the necessity of sufficiently long data series in order to achieve the reliability of the results. The same finding is frequently repeated in the research papers on entropy (many of which are quoted in the Introduction).

**Figure 2:**
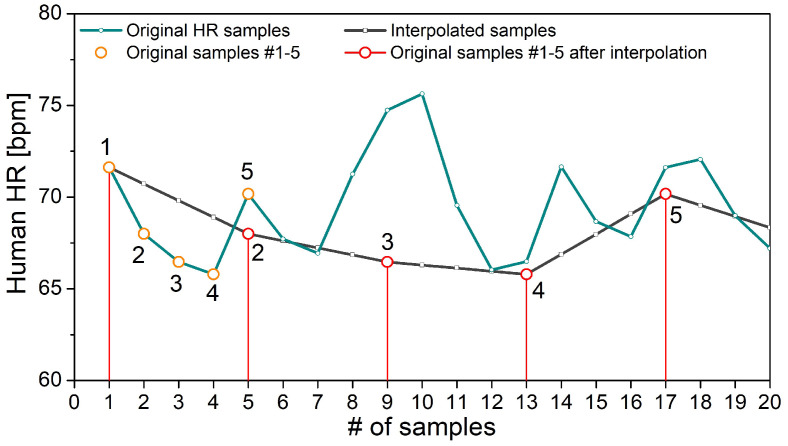
Heart rate (HR) samples from a healthy volunteer. The original and interpolated time series are shown in green and black, respectively. In both time series, the first five original samples are encircled. “#” denotes “number”.

Since one-minute recording is not sufficient, a trial was made to prolong the signals by a 5 Hz interpolation, presumably inspired by guidelines for power spectral density (PSD) estimation of cardiovascular signals [42]. If the one-minute record is resampled by 5 Hz, each one of the source samples (75 bpm = 1.25 Hz) would have been coupled with three statistically dependent ones, so, on average, 75% of the samples would be predictable results of interpolation. The outcome of such interpolation applied to the heart rate of a healthy volunteer is shown in Figure 2. However, the entropy level depends on the level of unpredictability of time series, and additional interpolated samples induce statistical dependency. By comparing the magnitude of the error made if the interpolated signals are used instead of the signals of sufficient length, we investigated whether the artificial signal prolongation is acceptable for entropy-based studies.

## 3. Results

### 3.1. Errors Estimated from Artificial Data

**Figure 3:**
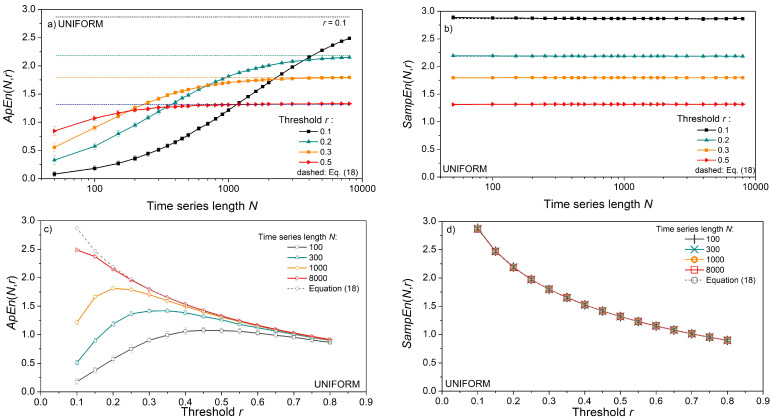
Entropy estimates for signals with uniform distribution. (**a**) *ApEn* as a function of time series length *N*; dashed lines show entropy evaluated by (18); (**b**) *SampEn* as a function of *N*; (**c**) *ApEn* as a function of threshold *r*; (**d**) *SampEn* as a function of *r*; *ApEn* is expressed as a mean ± standard deviation; *SampEn* is expressed as a mean value only, while its standard deviation is shown in Figure 4 for all three distributions.

The impact of time series length *N* and threshold *r* is shown in Figure 3. Entropy is estimated from the artificial data for vector size *m* = 2, and the results are presented as mean ± standard deviation (SD) in Figure 3a,c, and only as a mean in Figure 3b,d. We opted to show the results of uniform distribution as its entropy can be expressed in a closed form, (18), dashed lines. The results for Gaussian and exponential distributions are similar and, for this reason, omitted.

**Figure 4:**
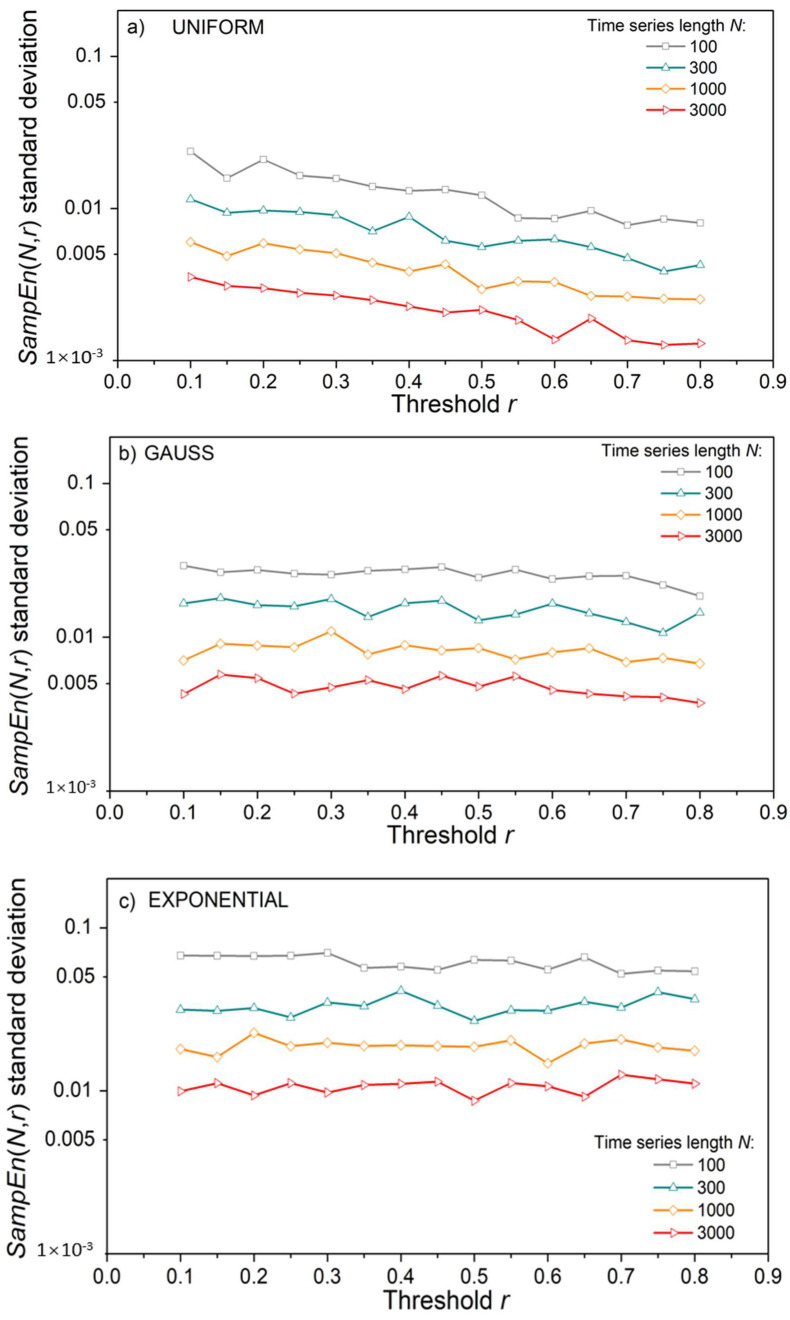
Standard deviation of *SampEn* as a function of threshold *r*, with a time series length *N* as a parameter; (**a**) Uniform time series distribution; (**b**) Gaussian distribution; (**c**) Exponential distribution.

*ApEn* estimates (Figure 3a,c) approach the theoretical values given by (18), but only for long time series and large threshold values. The estimates are well below the theoretical limit for thresholds *r* = 0.1 and 0.2, even in long data series (Figure 3a). Enlarging the threshold level to *r* = 0.5 is not sufficient for short time series of *N* = 100 and its entropy remains underestimated. On the other hand, estimated *SampEn* mean values in Figure 3b,d are in perfect accordance with theoretical values. Besides, in Figure 3d, there are five perfectly overlapping *SampEn* graphs, including the theoretical one, verifying the steady mean value of the estimates. These results are not new, as *SampEn* is designed to remove the *ApEn* inconsistencies. The standard deviation (SD) of *SampEn*, on the other hand, is larger than in *ApEn*. It would make the graphs in Figure 3b,d indistinctive, so it is presented separately in Figure 4.

**Figure 5:**
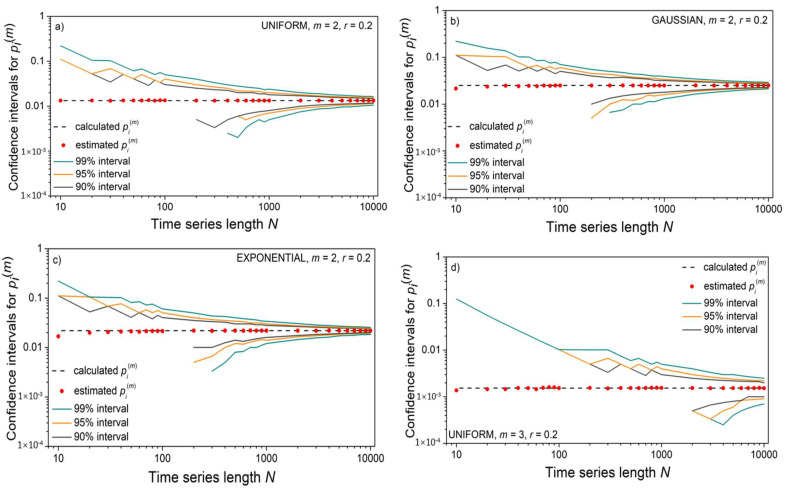

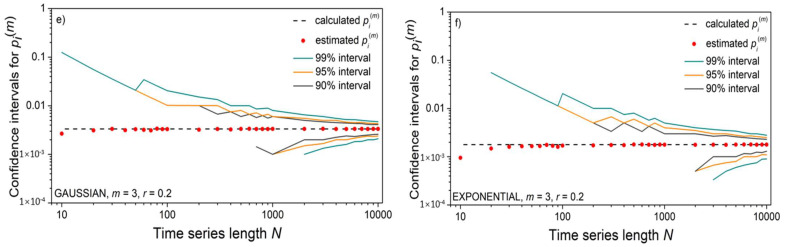
The confidence intervals for matching probability ${\hat{p}}_{i}^{\left(m\right)}\left(r\right)$; upper panels: template [−0.1 0.1], *m* = 2; lower panels: template [−0.1 0.1 0.6], *m* = 3; dashed lines: calculated $p_{i}^{\left(m\right)}\left(r\right)\;$ values, (15) and (17); circles: estimated ${\hat{p}}_{i}^{\left(m\right)}\left(r\right)\;$ values (4). Panels (**a**,**d**) correspond to uniform panels (**b**,**e**) to Gaussian and panels (**c**,**f**) to exponential distributions. Missing lower bound values are equal to zero.

Figure 4 shows that, contrary to the mean *SampEn* values, its standard deviation is affected by the time series length *N.* To show that it is an outcome of the relationship between the number of binary events (template matches) and the series length *N*, we estimated confidence intervals for an illustrative example of the *m* = 2 template [−0.1 0.1] and *m* = 3 template [−0.1 0.1 0.6], following a detailed reliability analysis of binary event probability estimates from [36,43]. For each *N*, 10^4^ time series were generated, and the corresponding mean probability values were both estimated and calculated, (4) and (15). The corresponding confidence intervals are shown in Figure 5 in the log–log scale. The values that are missing are equal to zero and cannot be shown as logarithms. Short time series have large confidence intervals, which causes an increased standard deviation of estimated mean values.

Another characteristic that might affect the stability of the results is zero-matches.

**Figure 6:**
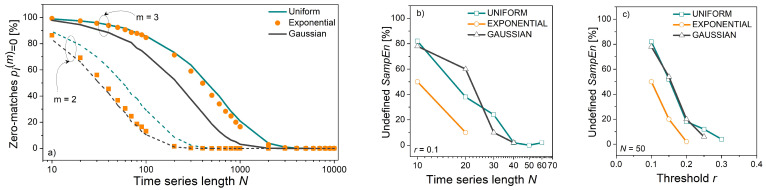
(**a**) Number of zero matches (in [%]); (**b**) Number of undefined *SampEn* estimates (in [%]) as a function of time series length *N*, for treshold *r* = 0.1; (**c**) Number of undefined *SampEn* estimates (in [%]) as a function of threshold *r*, for time series length *N* = 50.

Figure 6a shows the percentage of time series within which a template cannot find its match in *SampEn*, which is equivalent to finding its self-match in *ApEn*. The incidence of ${\hat{p}}_{i}^{\left(m\right)}\left(r\right)=0$ decreases below 10% for time series longer than *N* = 200 if *m* = 2, and longer than *N* = 2000 if *m* = 3. The number of zero-matches exceeds the number of other matching probabilities and disrupts their overall distribution. Moreover, if no pattern from the entire time series finds its match, *SampEn* is undefined because the logarithm of zero would appear in the (8). It can occur for low threshold values *r* and in short time series. The incidence of such events is shown in Figure 6b,c.

However, both *ApEn* and *SampEn* are prone to the same causes of instability, so it does not explain the difference in their estimates (Figure 3). The following results clarify this issue.

**Figure 7:**
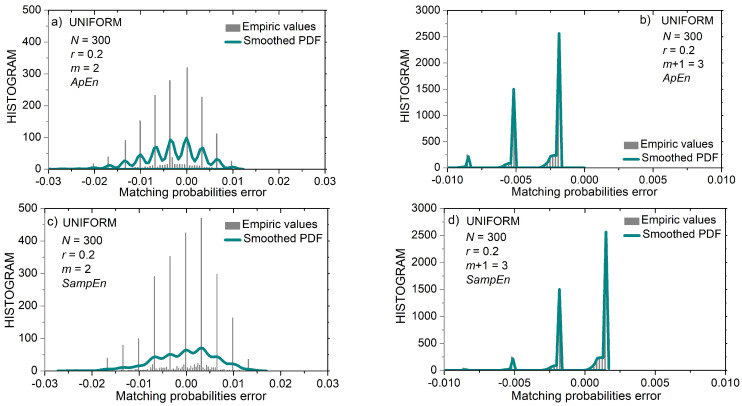
PDF of probability estimation error $\varepsilon_{Zi}^{\left(m\right)}\left(r\right)=p_{i}^{\left(m\right)}\left(r\right)-{\hat{p}}_{Zi}^{\left(m\right)}\left(r\right)\;,\;Z\in \left\{A,S\right\},$ for uniform time series of length *N* = 300 and for threshold *r* = 0.2. Black bars: empirc histogram; green lines: smoothed empiric PDF. (**a**) *ApEn*, *m* = 2; (**b**) *ApEn*, *m* = 3; (**c**) *SampEn*, *m* = 2; (**d**) *SanpEn*, *m* = 3.

**Figure 8:**
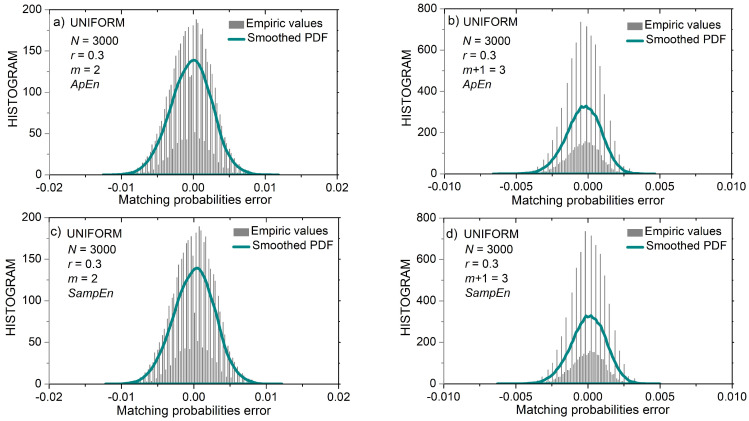
PDF of probability estimation error $\varepsilon_{Zi}^{\left(m\right)}\left(r\right)=p_{i}^{\left(m\right)}\left(r\right)-{\hat{p}}_{Zi}^{\left(m\right)}\left(r\right)\;,\;Z\in \left\{A,S\right\},$ for uniform time series of length *N* = 3000 and for threshold *r* = 0.3. Black bars: empirc histogram; green lines: smoothed empiric PDF. (**a**) *ApEn*, *m* = 2; (**b**) *SampEn*, *m* = 2; (**c**) *ApEn*, *m* = 3; (**d**) *SanpEn*, *m* = 3.

Figure 7 shows the distribution of matching probability errors $\varepsilon_{Zi}^{\left(m\right)}\left(r\right)=p_{i}^{\left(m\right)}\left(r\right)-{\hat{p}}_{Zi}^{\left(m\right)}\left(r\right)\;,\;\;Z\in \left\{A,S\right\},$ for short (*N* = 300) uniform time series. Figure 8 shows the same distribution but for longer (*N* = 3000) uniform time series. It can be seen that the error distribution of uniform time series is in part discrete and in part continuous.

The first part of the matching probability error is its estimated value (10). It is a discrete variable as it can have at most 1/(*N* − *m*) different values. The second part is the calculated matching probability. For uniform distribution, the calculated value is the same for most templates ((15), top line). It becomes continuous for the borderline amplitudes ($\left|x_{i}\right|\geq \sqrt{3}-r$). Observation of the x-axes in Figure 7a,c reveals that the distance between the discrete components (i.e., the distance between the discrete error values) is equal to 1/(*N* − *m*) ≈ 0.336, and Figure 8 shows that the same distance is 10 times smaller, in accordance with the ten times increased *N*.

Figure 7b,d show the error distribution for *m* = 3 templates. Since the matching probabilities are much lower than in the previous example, and the time series is short, the errors have three peaks showing that only 1, 2, or 3 matches were found for each template. It can be seen that Figure 7d is “shifted” along the *x*-axis by a value of 1/(*N* − *m*) ≈ 0.336 with respect to Figure 7b. Namely, due to excluded self-matches, the number of matches in the *SampEn* was reduced by one if compared to *ApEn*. Such a shift exists in all error distributions, but it is the most clearly visible in 7b and 7d.

An important feature of error distributions in Figure 8 is its symmetry, especially for *N* = 3000. The inspection of (12) reveals that estimated *SampEn* summands ${\hat{\Psi }}^{\left(m\right)}\left(r,N\right)$ can be expressed as:

(19) $${\hat{\Psi }}^{\left(m\right)}\left(r,N\right)=\ln\left(\sum_{i=1}^{N-m}p_{i}^{\left(m\right)}\left(r\right)-\sum_{i=1}^{N-m}\varepsilon_{Ai}^{\left(m\right)}\left(r\right)\right)$$

The second term within the logarithm is a summation of all the errors from the template matching procedure. Since error distribution is almost symmetric with respect to zero, the positive and negative error values mostly cancel each other. It holds even for the discrete distribution in Figure 7d, where one peak, but with more errors, cancels the remaining less exhibited peaks. Therefore, estimated summand ${\hat{\Psi }}^{\left(m\right)}\left(r,N\right)$ is almost error-free, as well as the corresponding entropy estimates.

In *ApEn* summand ${\hat{\Phi }}^{\left(m\right)}\left(r,N\right),$ the errors are not summed directly, but via a logarithmic function (11):

(20) $${\hat{\Phi }}^{\left(m\right)}\left(r,N\right)=\Phi^{\left(m\right)}\left(r,N\right)+\frac{1}{N-m+1}\cdot \sum_{i=1}^{N-m+1}\ln\left(1-\frac{\varepsilon_{Ai}^{\left(m\right)}\left(r\right)}{p_{i}^{\left(m\right)}\left(r\right)}\right)$$

so no error cancelation exists. On the contrary, errors are accumulating. Therefore, the improvement that *SampEn* induced is not only because the self-matching exclusion reduces the bias by shifting the error distribution along the error axis, thus increasing its symmetry with respect to zero, but essentially due to the method of *SampEn* estimation itself, as it implements this symmetry for error cancelation.

Only when estimated errors become very low (for time series length for which the confidence interval tightens), the error cancelation become irrelevant, and the errors also decrease in *ApEn* estimates.

**Figure 9:**
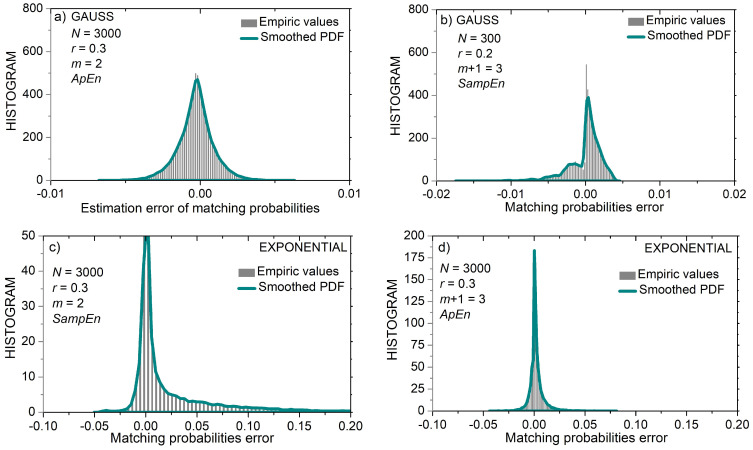
PDF of probability estimation error; (**a**) Gaussian distribution, *ApEn*, *m* = 2 and *N* = 3000; (**b**) Gaussian distribution, *SampEn*, *m* = 3 and *N* = 300; (**c**) Uniform distribution, *SampEn*, *m* = 2 and *N* = 3000; (**d**) Uniform distribution, *ApEn*, *m* = 2 and *N* = 3000.

Figure 9 shows examples of error distributions in Gaussian and exponential time series. The asymmetry of the error distribution in Figure 9b is due to the very low values of calculated matching probabilities: first, these probabilities are a product of *m* = 3 sample probabilities (15), and second, probability of samples from tail parts of Gaussian distribution can be very low, so the templates comprising them are not likely to find more than 2–3 matches. On the other hand, the resolution of the estimated matching probabilities is too coarse, 1/(*N − m*), to be compared with the fine granulation of the calculated probabilities. Thus, the PDF in Figure 9b is cut into intervals. Since the error in probability is expressed as $\varepsilon_{Si}^{\left(m\right)}\left(r\right)=p_{i}^{\left(m\right)}\left(r\right)-{\hat{p}}_{Si}^{\left(m\right)}\left(r\right)$, where the first term is continuous (15) and the second discrete (4), in the first interval, $\varepsilon_{Si}^{\left(m\right)}\left(r\right)\in [0,-\frac{1}{N-m})\;,$ the error is equal to $\varepsilon_{Si}^{\left(m\right)}\left(r\right)\;$ = $p_{i}^{\left(m\right)}\left(r\right)$; the second interval is defined as $\varepsilon_{Si}^{\left(m\right)}\left(r\right)\in [-\frac{1}{N-m}-\frac{2}{N-m})$ and the corresponding error values are $\varepsilon_{Si}^{\left(m\right)}\left(r\right)=p_{i}^{\left(m\right)}\left(r\right)-\frac{1}{N-m}$. The remaining segments follow the same pattern.

The increased time series length also increases the resolution of ${\hat{p}}_{Si}^{\left(m\right)}\left(r\right)$ estimates, and the error distribution becomes smooth and symmetrical, as shown in Figure 9b, for *m* = 2 and *N* = 3000.

Figure 9 also presents examples of matching probability error distribution estimated from exponential time series. PDFs are asymmetric, both in shape and with respect to zero. Positive errors are dominant, as the calculated probabilities exceed the estimated values.

Namely, the area below the exponential curve on the interval $\left[x_{i}-r,\;\;x_{i}\right]$ has large values that dominate the Equation (14), so that the calculated matching probabilities for the exponential distribution are higher than the pattern search can achieve (cf. Figure 1).

**Figure 10:**
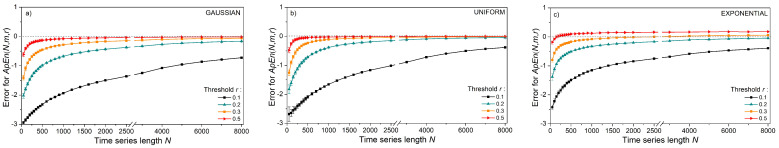

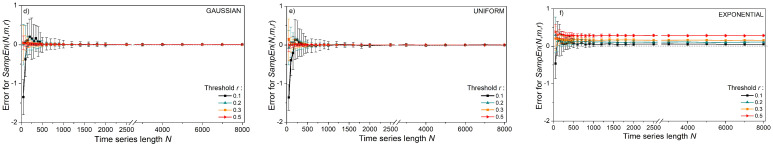
Errors in entropy estimates for template vectors of length *m* = 2; gray dashed line shows absence of errors; upper panels—*ApEn:* (**a**) Gaussian distribution; (**b**) uniform distribution; (**c**) exponential distribution; lower panels—*SampEn:* (**d**) Gaussian distribution; (**e**) uniform distribution; (**f**) exponential distribution.

Figure 10 presents the errors in entropy estimation for *m* = 2 and all three distributions—Gaussian, uniform, and exponential. *ApEn* errors (Figure 10a,b) are large and slowly converge towards zero for long data series. *SampEn* errors for the same distributions are different from zero for *N* < 500 and have a large standard deviation for *N* < 1500 (Figure 10d,e).

The skewness of the exponential time series causes an accumulation of positive errors in matching probabilities so that errors in entropy estimates do not converge to zero in both *ApEn* and *SampEn*. This further confirms the assumption that annulling the matching probabilities is the main reason for the stability of *SampEn*: if the errors cannot be annulled, the *SampEn* estimate is not consistent.

### 3.2. Errors in Experimental and Interpolated Time Series

The experimental SBP and PI beat-to-beat time series recorded from the laboratory rats comprised *N* = 8000 samples each. The mean PI value was 153.81 ± 12.17 ms, while the mean SBP value was 124.22 ± 18.22 mmHg. Each time series was divided into non-overlapping segments of length *N*. Entropy was estimated from each segment, and the results averaged, i.e., if *N* = 100, then 80 values are averaged, if *N* = 1000, then eight values are averaged.

**Figure 11:**
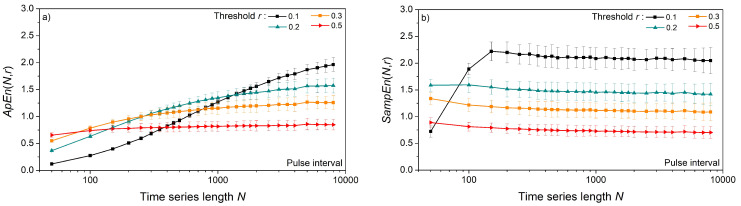

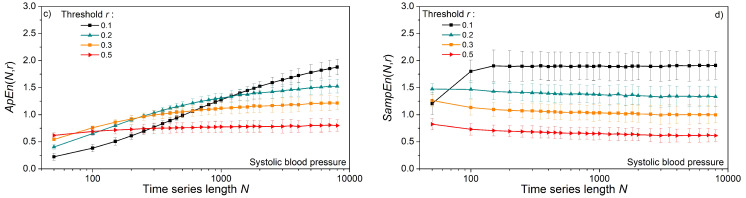
Entropy as a function of the time series length *N,* estimated from the signals of laboratory rats at baseline conditions, *m* = 2. (**a**) *ApEn* of pulse interval; (**b**) *SampEn* of pulse interval; (**c**) *ApEn* of systolic blood pressure; (**d**) *SampEn* of systolic blood pressure.

**Figure 12:**
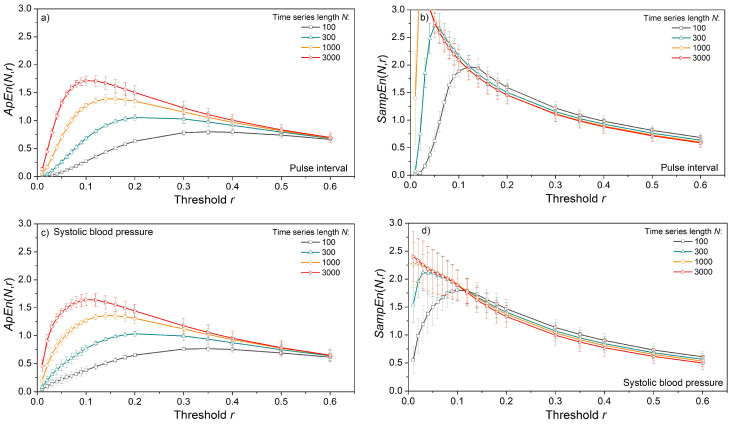
Entropy as a function of the threshold *r*, estimated from the signals of laboratory rats at baseline conditions, *m* = 2. (**a**) *ApEn* of pulse interval; (**b**) *SampEn* of pulse interval; (**c**) *ApEn* of systolic blood pressure; (**d**) *SampEn* of systolic blood pressure.

Figure 11 shows entropy as a function of time series length *N*, estimated from the pulse interval and systolic blood pressure of laboratory rats. For very long data series of length *N* > 5000, with a threshold *r* ≥ 0.2, the values of *ApEn* and *SampEn* are the same (Figure 11a,b). However, *SampEn* reaches a constant mean value for series as short as *N* = 500, while *ApEn* requires a much longer series. On the other hand, the standard deviation of *ApEn* is below the standard deviation of *SampEn*. This is a consequence of logarithms in *ApEn* since log-transformation is a classic method for increasing the stationarity of time series. The increased *SampEn* stability (smaller standard deviation) is reached with increased threshold values (Figure 12b,d). The entropy estimated from the SBP signals) is slightly lower than the entropy of the PI signal.

**Figure 13:**
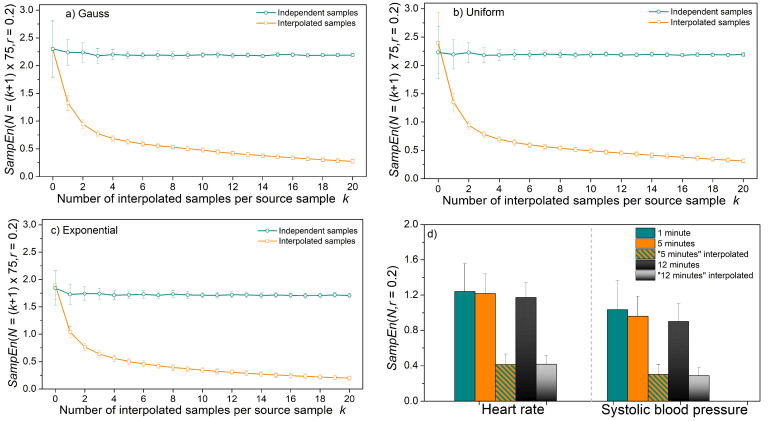
*SampEn* estimated from the original and interpolated time series; (**a**) time series with Gaussian distribution; (**b**) time series with uniform distribution; (**c**) time series with exponential distribution; (**d**) heart rate and systolic blood pressure time series recorded from healthy volunteers.

Figure 13 presents entropy estimated from the interpolated time series. The length of the initial time series was chosen to be *N* = 75 to correspond to one minute of the beat-to-beat cardiovascular data of a healthy volunteer. Then the interpolation was performed so that the number of samples is increased *k* + 1 times, *k* = 1, …, 20. Simultaneously, the time series of length *N* = *k*∙75 independent samples are generated. The results in Figure 13a–c for Gaussian, uniform, and exponential distributions show that entropy in interpolated, and therefore heavily statistically dependent time series, decreases.

The experiment is repeated for signals recorded from healthy volunteers with a mean heart rate equal to 73.27 ± 9.22 (bpm—beats per minute) and mean systolic blood pressure equal to 113.31 ± 11.5 (mmHg). Entropy was estimated from original recordings of duration 1 min, 5 min, and 12 min, showing no significant difference between the results. The entropy of interpolated time series is approximately three times lower than the entropy of the original time series.

Time series lengths of *N* = 75 are short and not too stable, as shown in the previous results, as well as in the difference of the mean values of the repeated experiments and the corresponding standard deviation for *k* = 0 (*N* = 75) in Figure 13a–c. However, the error magnitude is considerably smaller if *SampEn* were estimated from short time series than from interpolated time series.

As already mentioned, the interpolation does exist in the recommendations on heart rate variability, but for power spectral density estimation. However, the effects of interpolation for PSD and for entropy analysis are not the same. The interpolation in PSD is used to obtain equidistant samples, obligatory for Fourier transform. The signal power before and after the transform remains the same, regardless of the interpolation frequency (Parseval theorem [44]). Entropy, on the other hand, shows the irregularity and unpredictability of the time series. Lower entropy values correspond to the predictable data, and higher values to irregular, unpredictable data. Entropy estimates are based on the temporal similarity of template and matching vectors. If the interpolation is used, it induces temporal dependency and violates unpredictability.

## 4. Discussion and Conclusions

A key feature in the definition of *ApEn* and *SampEn* is the probability of pattern matching. *ApEn* is based on the sum of its logarithms that can be considered as informative content of the template. Formal *ApEn* interpretation is the difference between the average information carried by templates with incremental lengths, where the average is arithmetic.

*SampEn* is based on the logarithm of the average of matching probabilities, which can be considered as the information content of the average template of the observed time series. Formally, this can be considered a difference between the information carried by average templates with incremental lengths. Therefore, *ApEn* averages information estimated from matching probabilities, and *SampEn* estimates information from the average of matching probabilities.

*ApEn* inconsistencies have long been known and are usually explained by self-matching bias and a bias due to the different number of *m* and *m* + 1 patterns.

The analyses showed that the errors in entropy estimation are a consequence of the same reason as the quantization errors in signal digitization. The quants in estimating the matching probability are equal to 1/(*N* − *m*). For short signal lengths, the resolution of the estimated matching probability is too coarse to approximate its exact value, so the estimation error is large. This was confirmed with confidence intervals of estimated matching probabilities that are large for data lengths that are typical of most biomedical experiments (*N* up to 1000).

The quantization errors occur both in *ApEn* and *SampEn*. The reason for the difference in their consistency is the error distribution of the matching probabilities. This distribution is symmetric with respect to zero in most signals. The expression for *SampEn* sums these probabilities so that their errors cancel each other out, giving a consistent *SampEn* estimate. Disabling self-matching in *SampEn* shifts the error distribution along the error axis toward increasing symmetry, slightly improving error cancellation.

The resolution in matching probabilities increases simultaneously with the length of the time series. The quantization errors are then reduced, and *ApEn* and *SampEn* estimates are equalized. However, despite the stable *SampEn* means, its standard deviation is large and exceeds the standard deviation of *ApEn* even in very long data series. This is because logarithmic transformation is a classic method for smoothing signals. Since each estimated matching probability is logarithmically transformed, their standard deviation is attenuated.

Skewed distributions, such as exponential, yields asymmetric matching probabilities and induce a systematic error in *SampEn* estimates.

It follows that both *ApEn* and *SampEn* have advantages and disadvantages. For time series with symmetrical PDF, *SampEn* is stable in mean even for short data series, but with large variance. For the same series, *ApEn* yields erroneous values but with a very small standard deviation. For very long data series, *ApEn* is better as it gives the same estimates as *SampEn*, but with a significantly smaller standard deviation than in *SampEn*. For asymmetric PDF, *SampEn* induces errors in estimates while *ApEn* yields the correct value, provided that the time series is sufficiently long.

Entropy estimated from experimental data reveals that time series should not be shorter than *N* = 500 and that the threshold should not be below 0.3. It is also shown that interpolation cannot be used for artificial time series lengthening, as it distorts the very property of signal that is measured by entropy.

Further research will follow the idea of non-uniform quantization implemented for speech coding in digital transmission systems: to develop variable threshold levels adapted to the amplitude distributions of the observed signals. Templates containing large but rare amplitudes from the tails of the signal density function would be associated with a higher threshold. A template containing smaller but more likely amplitudes would be associated with a smaller threshold. Such a procedure would definitely give a smaller average error in estimating the probability of a match, but it remains to be seen whether it would give a reliable estimate of entropy.

## Data Availability

Restrictions apply to the availability of these data. Data were obtained from grant III41013 and are available from PI (N.J.-Ž.), with the written permission of all the participants. Data from healthy volunteers are not publicly available due to the privacy constraints agreed upon within the signed informed consent.
